# Sex differences in vitamin D and behavioral profiles among children with allergic diseases

**DOI:** 10.1002/fsn3.3505

**Published:** 2023-06-15

**Authors:** Chia‐Jung Li, Ling‐Sai Chang, Mindy Ming‐Huey Guo, Liang‐Jen Wang, Ho‐Chang Kuo

**Affiliations:** ^1^ Department of Child and Adolescent Psychiatry Kaohsiung Chang Gung Memorial Hospital and Chang Gung University College of Medicine Kaohsiung Taiwan; ^2^ Department of Pediatrics Kaohsiung Chang Gung Memorial Hospital and Chang Gung University College of Medicine Kaohsiung Taiwan; ^3^ Kawasaki Disease Center Kaohsiung Chang Gung Memorial Hospital Kaohsiung Taiwan

**Keywords:** allergic rhinitis, asthma, atopic dermatitis, IgE, vitamin D

## Abstract

Previous studies have suggested that vitamin D has a protective effect on allergic diseases, while an individual's sex may have a moderating effect on the relationship between vitamin D and allergic‐related immunity. This study aimed to determine the role of vitamin D in children with coexisting allergic diseases in the context of sex differences and to explore the behavioral profiles of these patients. We recruited a total of 103 children with atopic diseases and divided them into four groups: males with one allergic disease (MA1, *n* = 20), males with two or more allergic diseases (MA2, *n* = 26), females with one allergic disease (FA1, *n* = 30), and females with two or more allergic diseases (FA2, *n* = 27). We measured serum calcium levels using the colorimetric method and serum 25‐OH vitamin D total levels using electrochemiluminescence immunoassay. We found that MA2 had significantly lower vitamin D levels than MA1 and FA2. The levels of IgE were negatively correlated with vitamin D in females, whereas the levels of IgE were not significantly correlated with vitamin D in males. Furthermore, serum IgE was significantly correlated with children's adaptive skills, and different sexes were associated with different aspects of adaptive skills. Our findings suggest a protective role of vitamin D in the development of one allergic disease against the coexistence of allergic diseases in males, as well as extend the evidence for sex differences in immunity by demonstrating a sex‐different correlation between IgE and vitamin D and the relationship between IgE and children's adaptive skills.

## Graphical abstract

This study revealed that males with two or more allergic diseases had significantly lower vitamin D levels than male with one allergic disease. We suggest a protective role of vitamin D in the development of one allergic disease against the coexistence of allergic diseases in males.

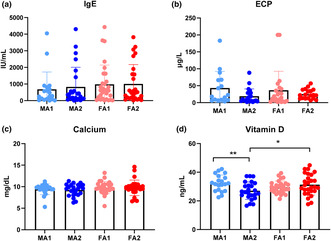

## 1 INTRODUCTION

The incidence of allergic diseases, including atopic dermatitis, allergic rhinitis, and asthma, has increased more than 10–20 times in recent decades. In Taiwan, children and adolescents have a significantly high prevalence of these allergic diseases, which has made the study of children with allergiesmore feasible and accessible (Lee et al., 2004). Children with atopic dermatitis (AD) account for about 21.6% of all children in the national insurance database survey (Woon et al., 2013); about 25% have asthma, while allergic rhinitis affects almost 50% of children in Taipei City. In addition, children with more than one allergic disease and coexistence status commonly appear prevalent with age. One British study demonstrated that the prevalence of multiple allergic diseases is 11% for children aged 2–15 years (Gupta et al., 2004). Furthermore, atopic dermatitis, asthma, and allergic rhinitis often co‐occur in the same individual. A Korean study revealed that the percentage of having any two allergic diseases among atopic dermatitis, asthma, and allergic rhinitis is between 2.5% and 8.7% (Hong et al., 2012). Some studies revealed an even higher prevalence rate of more than 20% in pediatric patients with coexisting allergic diseases (Arabkhazaeli et al., 2015). The coexistence of allergic conditions indicates more complicated allergic conditions, which are often more poorly controlled.

The mechanism of vitamin D in immunity has gradually been established in recent decades (Hemamy et al., 2020; Mirzakhani et al., 2015). Vitamin D affects innate immunity by inducing antimicrobial peptides and strengthening the physical epithelial barrier, as well as adaptive immunity via a direct effect on T‐cell activation and related cytokines (Benson et al., 2012). Most of these immune responses were mediated by vitamin D binding proteins and receptors, which have been identified in virtually all cells of the immune system, including T and B cells, macrophages, and dendritic cells. The multifactual ability impacts immune cells, modulates inflammatory responses, and prevents the predisposing infection, thus making vitamin D a potential factor in reducing the severity of allergic diseases. Several epidemiological studies have found that low vitamin D levels are associated with allergy prevalence, and some have further demonstrated that vitamin D reduced the exacerbation and hospitalization of allergic diseases, suggesting that vitamin D has a protective effect on allergic diseases (Brehm et al., 2009; Ogeyingbo et al., 2021; Turkeli et al., 2016). A meta‐analysis that focused on children with atopic dermatitis found that they tend to have low levels of serum 25(OH)D and are at a higher risk of developing vitamin D insufficiency (Fu et al., 2022). Another recently published meta‐analysis showed that low levels of vitamin D in mothers are also associated with a greater risk of their infants developing food allergies (Psaroulaki et al., 2023).

Like vitamin D, calcium is a dietary supplement widely used in Europe as a remedy for allergic pruritus or erythema. However, the efficacy of calcium supplementation on allergy remains controversial (Hildebrand et al., 2019; Matysiak et al., 2017), and the exact mechanism remains unclear. Currently, the link between calcium and allergic diseases has primarily been derived from studies on dietary supplements, as noted above, or from asthma studies, which have shown better control in airway conditions by enhanced calcium homeostasis due to the regulation of smooth muscle tone (Weiss, 1990; Yarova et al., 2015). The role of vitamin D in allergic diseases in recent decades has aroused curiosity with regard to whether calcium has a similar role or whether it is dependent or independent of vitamin D. To the best of our knowledge, research into the relationship among vitamin D, calcium, and allergy has mainly focused on a specific allergic disease. However, while the effects of vitamin D on immunity are multifaceted and systemic, few studies have targeted the coexistence of allergic diseases; therefore, exploring the relationship between these biomarkers and the coexistence of allergic diseases is of great significance.

A growing body of research has shown that sex is an important variable in immunity, including the allergy field. Epidemiological data have revealed a strong sex disparity in asthma prevalence, severity, hospitalizations, and mortality. One previous study that targeted allergic rhinitis also found sex differences in the prevalence (Akinbami et al., 2012; Moorman et al., 2012). In experimental studies, estrogen has proven to influence immune responses at multiple levels, involving the development of both B and T cells and promoting Th cell polarization and IgE production, as well as mast cell and basophils degranulation (Bouman et al., 2005; Cunningham & Gilkeson, 2011). Like with vitamin D, a recent review indicated that sex differences in vitamin D were found at multiple levels, including the genetic expression of VDR and the synthesis and metabolism of vitamin D. With regard to allergic immune responses, one study found that vitamin D affects TNF‐α and IL‐6 concentration in female's adipose tissue, but not in males. More detailed studies are needed to clarify the sex differences in vitamin D in the context of allergic‐related immunity.

Many studies have emphasized the association between behavioral problems and allergies, suggesting children with allergies are more likely to experience inattention and both externalized and internalized behavioral problems. Furthermore, a considerable psychosocial impact can be experienced by children, leading to reduced life satisfaction and difficulties in adaptation (Haanpaa et al., 2018; Park et al., 2011). However, not all studies have supported the positive association between allergic disease and behavioral and adaptive problems. One possible reason may be that studies targeting the matter often encountered limitations in controlling complex biological and psychosocial confounders (Calam et al., 2003; Chang et al., 2013; Oades et al., 2010). From a biological viewpoint, chronic inflammatory responses in the body can impact brain function, leading to changes in behaviors and maladaptation. Parental stress regarding the disease is also believed to be a crucial part associated with children's adaption and behavioral problems.

To better understand the role of vitamin D in allergic diseases, we conducted a cross‐sectional study to investigate the relationship among vitamin D, calcium, and allergic‐related biomarkers (IgE and eosinophil cationic protein [ECP]) among patients with allergic diseases, disaggregated by sex and single or coexistence status of allergic disease. We also explored whether coexistence status and sex factors impacted children's behavioral profiles and parental stress.

## 2 MATERIALS AND METHODS

### 2.1 Study participants

This study was approved by the local Ethics Committee of the Chang Gung Memorial Hospital (reference number 201900465A3). Written informed consent was obtained from all the enrolled children and their parents or guardians. We recruited a total of 103 patients with atopic diseases from the Department of Pediatrics, Kaohsiung Chang Gung Memorial Hospital, Taiwan, or communities near the hospital. Patients who had one or more atopic diseases (allergic rhinitis, asthma, atopic dermatitis, or urticaria) were recruited by a properly trained allergist clinician.

### 2.2 Laboratory assessments

We collected blood samples from participants in the study during their visit, and measurements were carried out in the central laboratory of CGMH. The Pharmacia CAP system (Pharmacia & Upjohn Diagnostic AB) was adopted to determine levels of IgE and ECP concentration. Serum calcium was measured using the colorimetric method and serum 25‐OH vitamin D total using electrochemiluminescence immunoassay (ECLIA).

### 2.3 Clinical measurements

The Swanson, Nolan, and Pelham IV Scale (SNAP‐IV) consists of a 26‐item questionnaire and is used to evaluate ADHD symptoms and severity (Bussing et al., 2008). The 26 items include 18 involving ADHD symptoms (9 for inattention and 9 for hyperactivity and impulsivity) and 8 that involve oppositional defiant disorder symptoms. Each item is scored on a 3‐point Likert scale. The Chinese versions of the SNAP‐IV parent form (Gau et al., 2008) have been reported as having satisfactory reliability and concurrent validity.

### 2.4 Adaptive Behavior Assessment System‐II (ABAS‐II)

The traditional Chinese version of the teacher form of the ABAS‐II (Hsu et al., 2021), originally developed by Harrison and Oakland, was used to measure participants' overall adaptive development. The ABAS‐II measures four composite scores for the following skill categories: (a) conceptual, (b) social, (c) practical, and (d) general adaptive skills. The general adaptive composite score is an overall standard score that summarizes an individual's adaptive functioning across all skill areas.

### 2.5 Parental stress

Caregivers' parenting function was assessed using the Parenting Stress Index–Short Form (PSI‐SF). The PSI‐SF, a widely adopted self‐administered questionnaire for evaluating parental stress, includes 36 items (rated on a 5‐point Likert scale) and stems directly from the full‐length 120‐item Parenting Stress Index (PSI) test (Loyd & Abidin, 1985). The PSI‐SF provides scores in the following subscales: (a) parental distress, (b) parent–child dysfunctional interaction, and (c) difficult child. The three subscores are added together to yield a total parenting stress score (Haskett et al., 2006). Studies have reported that the Chinese version of the PSI‐SF is a reliable assessment tool for identifying parenting stress that requires intervention in clinical practices (Wang et al., 2021).

### 2.6 Statistical analysis

We utilized the statistical software package SPSS, version 16.0 (SPSS Inc.), for data analysis. Two‐tailed *p*‐values < .05 were considered statistically significant. Variables are presented as either the mean (standard deviation) or frequency. The normality of distribution of continuous data was examined using the Shapiro–Wilk test. We found that behavioral data (SNAP‐IV, ABAS‐II, and PSI‐SF) and levels of vitamin D were normally distributed. However, IgE, ECP, and calcium levels violated normal distribution. We further performed log transformation in order to obtain the normal distributed data of IgE, ECP, and calcium.

We applied Chi‐square test or Fisher's exact test to compare the ratios of atopic diseases between ADHD patients and controls. We adopted one‐way analysis of variance (ANOVA) to compare clinical characteristics, IgE, ECP, calcium, and vitamin D among the study's four groups of children (males with one allergic disease [MA1], males with two or more allergic diseases [MA2], females with one allergic disease [FA1], and females with two or more allergic diseases [FA2]). The potential differences in the log‐transformed data of IgE, ECP, and calcium among the four groups were also examined using ANOVA. We used LSD post‐hoc analyses for multiple‐comparison adjustments. Finally, Pearson correlation was used to examine the relationship among IgE, ECP, calcium, and vitamin D.

## 3 RESULTS

This study recruited 103 children with allergic diseases (mean age: 5.9 years old, 57.1% male). Of those, 62 (60.2%), 58 (56.3%), 39 (37.9%), and 2 (1.9%) patients suffered from allergic rhinitis, asthma, atopic dermatitis, and urticaria, respectively. Of all participants, 50 (48.5%) suffered from one allergic disease, and 53 (51.5%) had two or more allergic diseases. The characteristics of the four participant groups (males with one allergic disease [MA1, *n* = 20], males with two or more allergic diseases [MA2, *n* = 26], females with one allergic disease [FA1, *n* = 30], and females with two or more allergic diseases [FA2, *n* = 27]) are summarized in Table 1.

**TABLE 1:** Characteristics of four groups of children (males with one allergic disease [MA1], males with two or more allergic diseases [MA2], females with one allergic disease [FA1], and females with two or more allergic diseases [FA2]).

	MA1 (*n* = 20)	MA2 (*n* = 26)	FA1 (*n* = 30)	FA2 (*n* = 27)	Statistics	*p*‐value
Age (years), mean (SD)	4.9 (1.3)	6.5 (2.0)	6.2 (2.6)	5.9 (3.0)	1.931	.129
Allergy diseases						
Allergic rhinitis	8 (40%)	23 (88.5%)	5 (16.7%)	26 (96.3%)	54.344	<.001^*^
Asthma	6 (30%)	22 (84.6%)	11 (36.7%)	19 (70.4%)	21.232	<.001^*^
Atopic dermatitis	6 (30%)	10 (38.5%)	13 (43.3%)	10 (37%)	0.941	.831
Urticaria	0 (0%)	1 (3.8%)	1 (3.3%)	0 (0%)	1.868	.846
SNAP‐IV	20.4 (7.3)	24.3 (11.04)	26.15 (8.5)	18.67 (12.2)	1.136	.349
ABAS‐II						
General	105.4 (9.6)	97.4 (12.6)	101.5 (10.2)	103.14 (12.2)	1.003	.399
Conceptual	106 (5.6)	98.7 (11.5)	101.7 (10.6)	102.6 (13.9)	0.709	.551
Social	105 (13.8)	98.8 (10.8)	103.41 (11.3)	102.9 (13.8)	0.575	.634
Practical	106.14 (9.1)	98.07 (15.8)	100.06 (9.1)	103.9 (10.1)	1.072	.369
PSI‐SF						
Parental distress	29 (8.9)	31.8 (8.5)	31.2 (9.6)	28.8 (6.9)	0.421	.739
Parent–child dysfunctional interaction	21.7 (5.8)	22.8 (7.2)	21.9 (7.9)	23.2 (7.6)	0.118	.949
Difficult child	25.1 (4.9)	28.9 (6.8)	25.7 (8.6)	27.9 (6.6)	0.810	.494

*Note*: Data are expressed as mean ± SD or *n* (%).

Abbreviations: ABAS‐II, Chinese Adaptive Behavior Assessment System–2nd Ed. for Children; PSI‐SF, Parenting Stress Index–Short Form; SNAP‐IV, The Swanson, Nolan, and Pelham IV Scale.

* *p* < .05.

**FIGURE 1:**
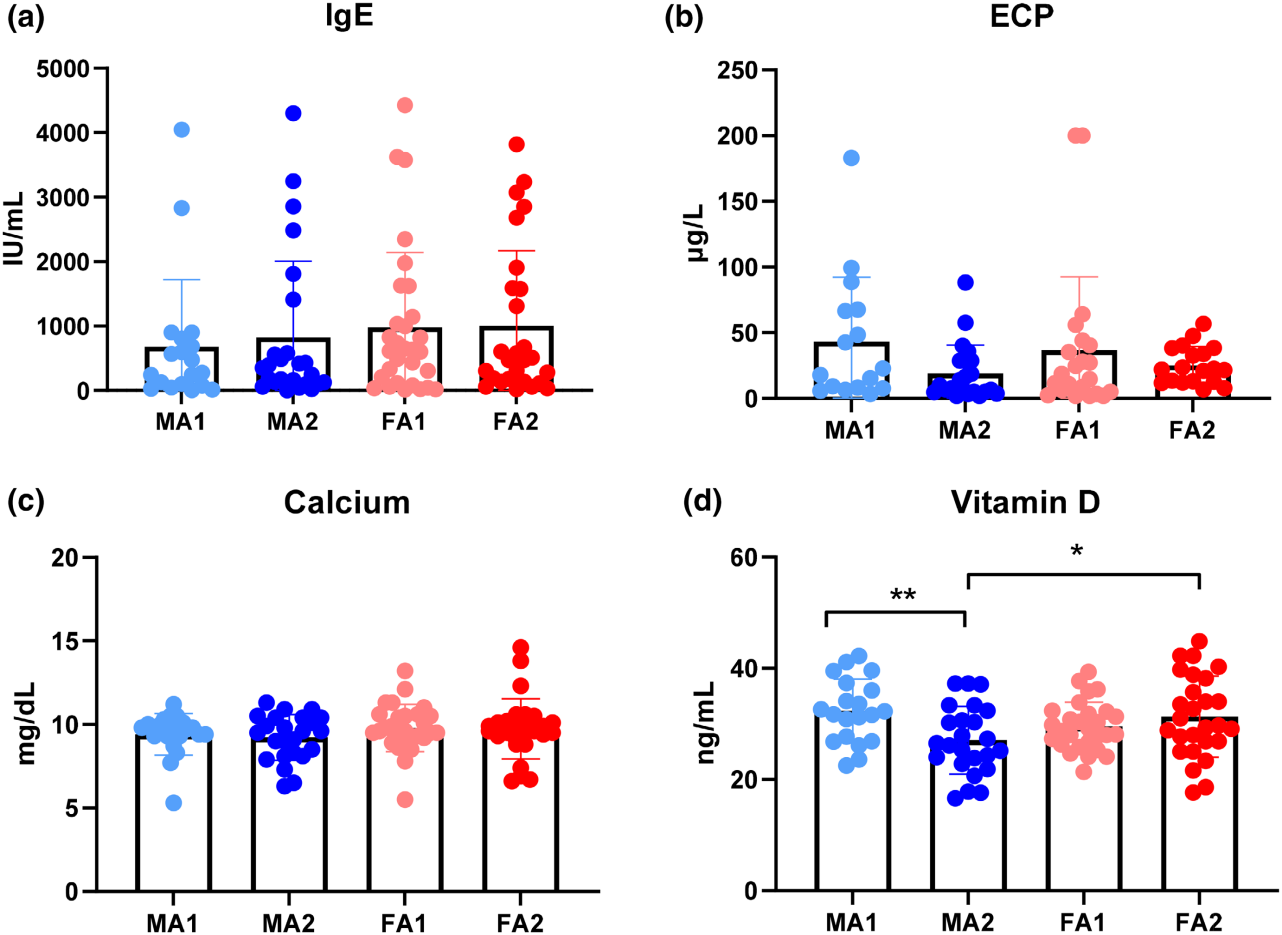
Plasma levels of IgE (a), ECP (b), Calcium (c) and Vit D (d) among four groups children (males with one allergic disease [MA1], males with two or more allergic diseases [MA2], females with one allergic disease [FA1], and females with two or more allergic diseases [FA2]. *p < .05, **p < .01.

In Table 1, we observed no significant differences in the distribution in any of the behavioral profiles (SNAP‐IV, ABAS‐II, and PSI‐SF) among the four groups (*p* > .05). The plasma levels of IgE, ECP, calcium, and vitamin D among the four groups are displayed in Figure 1. The IgE, ECP, and calcium levels did not differ significantly among the four groups. However, the vitamin D levels revealed significant group differences (*F* = 3.46, *p* = .019). The log‐transformed data of IgE, ECP, and calcium were further used for ANOVA testing, where no significant differences were observed in the IgE, ECP, or calcium levels among the four groups.

**FIGURE 2:**
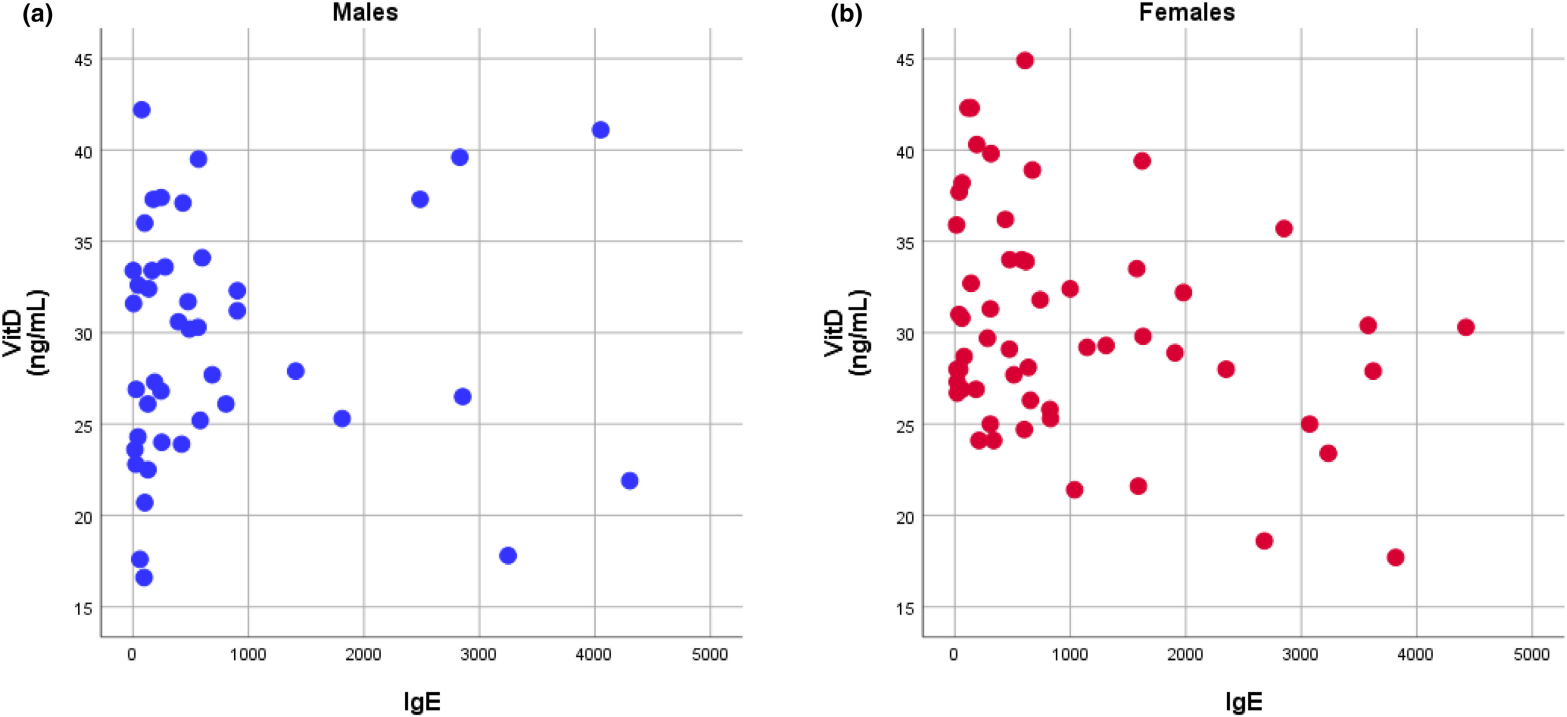
The relationships between IgE and Vit. D in males (a) and females (b), respectively. The levels of IgE were not significantly correlated to Vit. D in males (*r* = .055, *p* = .728), and the levels of IgE were negatively correlated to Vit. D in females (*r* = −0309, *p* = .022).

Figure 2 demonstrates the relationships between IgE and vitamin D in males and females, respectively. The levels of IgE were not significantly correlated with vitamin D in males (Figure 2a, *r* = .055, *p* = .728), while the levels of IgE were negatively correlated with vitamin D in females (Figure 2b, *r* = −.309, *p* = .022). Among males, the IgE levels were significantly correlated with the practical adaptive skills of ABAS‐II (*r* = −.514, *p* = .017). Among females, the IgE levels were significantly correlated with the conceptual ABAS‐II (*r* = −.415, *p* = .020) and the general adaptive skills (*r* = −.362, *p* = .045). Calcium and vitamin D levels were not significantly associated with the behavioral profiles of children with allergy diseases.

## 4 DISCUSSION

Our results revealed that boys with two or more allergic diseases had significantly lower vitamin D levels compared with boys with one allergic disease and girls with two or more allergic diseases, suggesting a negative association between vitamin D level and the risk of coexistence of allergic diseases, which was prominent among boys. Furthermore, our data demonstrated that the patterns of the relationship between IgE and vitamin D differed between boys and girls. In boys, the level of IgE was not significantly correlated with vitamin D, while IgE level was negatively correlated with vitamin D in girls.

Previous studies on vitamin D metabolites have demonstrated potential differences in metabolic patterns between genders among patients with autoimmune diseases. For instance, Kragt et al. (2009) found that higher levels of 25(OH)D were linked to a reduced risk of developing multiple sclerosis (MS) and MS‐related disability, but only in women. Similarly, a case–control study by Barnes et al. (2007) found that women with MS had higher levels of both 25(OH)D and 1,25(OH)2D3 than men with MS. Although the exact mechanism behind these findings is unknown, they suggest that sex differences may affect the way vitamin D is metabolized in people with MS. Furthermore, studies have demonstrated that in females with MS, vitamin D has a more prominent influence on the regulation of T cells and immune cytokines compared to males, which expresses a stronger reactivity in immune response (Wierzbicka & Oczkowicz, 2022). Despite strong evidence of sex differences in vitamin D being found in relation to autoimmune diseases, evidence with regard to sex differences in vitamin D in allergic diseases remains scarce. Most discussions have been limited to sex differences in allergic immune responses and experimental studies. Estrogen, based on its concentration, was proven to mediate Th1/2 balance and induct proinflammatory cytokines, for example, IL‐4, which is related to an allergic reaction (Dupuis et al., 2021; Straub, 2007). In various human and rat tissues, estrogen was demonstrated to enhance the expression of vitamin D receptors (Escaleira et al., 1993; Liel et al., 1999). Furthermore, in the animal model, estrogen was found to increase vitamin D accumulation, leading to more potent anti‐inflammatory responses in females (Spach & Hayes, 2005). The present study has provided data among vitamin D, sex differences, and allergic diseases by revealing that vitamin D level is lower in males with coexistence allergic disease, compared with males with only one allergic disease. These data suggest that vitamin D may have a role in protecting against the development of one to the coexistence of two or more allergic diseases, but only among the male group. This hypothesis is also in line with an animal study that demonstrated in male mice that the group with vitamin D deficiency showed a significant contact hypersensitivity response compared to the males with normal vitamin D levels (Malley et al., 2009).

Our findings suggest that vitamin D levels are associated with mechanisms that contribute to allergic comorbidity, which is characterized by a systemic inflammatory state involving multiple organs. We propose two possible explanations. The first is the link between vitamin D and atopic march. Many pediatric patients with allergies follow a special atopic trajectory starting from atopic dermatitis, allergic rhinitis, and then asthma in their early years, which is known as the atopic march. Notably, patients often follow the trajectory with two or more allergic diseases. New insights have pointed to the skin barrier defect being the main cause of atopic dermatitis and which is believed to be responsible not only for atopic dermatitis but also for later allergic sensitization, such as asthma and allergic rhinitis (Bantz et al., 2014; Spergel, 2010; Weidinger & Novak, 2016). The lack of dermal integrity, resulting from the reduction in lipids and abnormal keratinization, permits the penetration of allergens through the skin and further initiates TH1/2 immune responses, resulting in atopic dermatitis and subsequent asthma and allergic rhinitis. Skin and vitamin D levels have a bi‐directional relationship since the skin is where vitamin D biosynthesis occurs, as well as the target organ for vitamin D to affect immunity. Studies have also proven the role of vitamin D in the homeostasis of the skin's barrier function through its effects on epithelial cell proliferation, differentiation, apoptosis, and modulation of the immune system (Schwalfenberg, 2011). The second explanation is the link between vitamin D and the microbiome. Emerging evidence suggests that vitamin D may play an intermediary role in the microbiome, which is crucial for the maturation and development of both Th1 and T regulatory pathways involved in suppressing immune responses. By regulating antimicrobial peptide expression and maintaining the stability of gut mucosa, vitamin D and VDR together maintain the balance of gut microbiota (Akimbekov et al., 2020; Murdaca et al., 2021). People with vitamin D inadequacy and alteration of VDR signaling are associated with damaged gut mucosa, resulting in microbiome dysbiosis. Furthermore, microbiota may regulate VDR expression, resulting in an intertwined interaction between vitamin D and microbiota (Kong et al., 2008; Murdaca et al., 2021; Reich et al., 2014).

Data from human studies focusing on the relationship between vitamin D and serum IgE are limited. Two studies targeting pediatric patients with asthma revealed a significant inverse correlation between vitamin D3 levels and serum IgE (Hatami et al., 2014; Mohammadzadeh et al., 2020), but another study reported no significant correlations in this matter (Chinellato et al., 2018). A British study with a large sample revealed a nonlinear, U‐shaped association between vitamin D and IgE: little variation in IgE when vitamin D was between 12 and 48 ng/mL, while IgE increased significantly when vitamin D levels were either below 10 ng/mL or over 50 ng/mL (Hypponen et al., 2009). An animal study demonstrated that vitamin D supplementation resulted in an increase in inflammatory cytokines and serum IgE (Matheu et al., 2003). The above studies demonstrate that vitamin D can have both a beneficial and a detrimental effect on immunity. The present study revealed that only the female group demonstrated a negative association between vitamin D and IgE, while no significant association was found in the male groups, thus indicating sex as a variable in the relationship between vitamin D and IgE among preschool‐age children. The result is somewhat in line with a birth cohort study that recruited 275 children and showed that children had different age and sex patterns in cytokine responses, total IgE levels, and blood eosinophilia in early childhood. By the age of 1 year, boys were more likely to have increased total IgE levels and allergic sensitization. By the age of 3 years, boys tended to present enhanced Th1 and Th2 cytokine responses and increased total IgE and eosinophilia (Uekert et al., 2006).

Although allergic diseases are generally not life threatening, they can influence children's behaviors and adaptations, as well as parental stress. These impacts result from both psychological and biological aspects. Psychologically, children may experience psychological adjustment to acute exacerbation or chronic status of allergic disease; biologically, allergic‐related inflammatory cytokines can affect emotional and behavioral regulations. The present study found that serum IgE was significantly correlated with children's adaptive skills, as well as that different sexes were associated with different aspects of adaptive skills. Studies have shown that IgE levels reflect increased psychological and biological stress (Loureiro & Wada, 1993; Stephan et al., 2004; Wright et al., 2004), which affects children's adaptive skills. In addition, elevated IgE levels affect such brain functions as learning and memory, which may also impact daily adaptation and quality of life (Kohman & Rhodes, 2013). However, we need more studies to clarify the underlying mechanism in this association.

The present study failed to find significant differences in either children's behavioral profiles or their parental stress among the four groups of children. The finding implies that the coexistence of allergic diseases does not necessarily mean that parental stress and children's behavior are more problematic or complex; in other words, there is no “dose‐dependent” relationship between parental stress, children's behavioral problems, and number of allergic diseases. This finding somewhat contradicts the impression that caring for children with coexisting allergic diseases involves a greater psychosocial burden and more tasks than for children with only one allergic disease, resulting in greater parental stress. Studies focusing on allergic diseases and children's behaviors have demonstrated that behavioral problems correlate with clinical manifestations, severity of allergic diseases, and caregiver stress (Lee et al., 2014; Yamaguchi et al., 2021). Many of these studies are limited to a specific allergic disease, but not multiple allergic diseases. A cohort study found a dose‐dependent association between children with multiple allergic diseases and a higher risk of developing internalizing problems like anxiety and depression (Nanda et al., 2016). However, the study did not address externalizing and adaptive behaviors, which were of interest and demonstrated no dose‐dependent association between multiple allergic disease states in our study. On the other hand, since parental stress and children's behavioral problems and adaption are often interrelated when observed from that perspective, it is reasonable that neither children's behavioral profiles nor parental stress showed significant differences among the four studied groups.

Our study has both strengths and limitations. Children with allergic diseases often have more than one allergic disease, so our study focused on coexistence and explored its relationship with allergic‐related biomarkers, children's behavioral profiles, and parental stress. In addition, our study extended the evidence for sex differences in immunity by demonstrating a sex difference correlation between IgE and vitamin D, as well as the relationship between IgE and children's adaptive skills. The study's limitations are as follows: First, our study samples are not randomly assigned and may not accurately represent the intended population. Second, this is a cross‐sectional study, so we could not address the causality of our findings, or did we record the duration and acute/chronic state of allergic diseases the children were in. Third, serum vitamin D level is sensitive to race, age, and residual area; in the absence of control groups, we were unable to analyze whether any other variables influence our results. Fourth, we chose total IgE rather than specific IgE as one of the allergic‐related biomarkers since our study aimed to examine factors associated with the coexistence of allergic disease in children rather than with the diagnosis of specific allergic diseases. However, the poor specificity of total IgE limited our analysis to specific allergic diseases.

## 5 CONCLUSION

We observed sex differences in the relationship between vitamin D levels and the coexistence risk of allergic disease. A sex‐specific pathway was also noted in the relationship between vitamin D and IgE in allergic pediatric patients. Our data showed that neither children's behavioral profiles nor parental stress was associated with the increasing number of allergic diseases. More research is needed to explore the role of vitamin D in allergic diseases in the context of sex differences.

## AUTHOR CONTRIBUTIONS

**Chia‐Jung Li:** Data curation (equal); formal analysis (equal); writing – original draft (equal). **Ling‐Sai Chang:** Data curation (equal); investigation (equal); project administration (equal); resources (equal). **Mindy Ming‐Huey Guo:** Conceptualization (equal); investigation (equal); methodology (equal); resources (equal). **Liang‐Jen Wang:** Conceptualization (equal); formal analysis (equal); investigation (equal); supervision (equal); visualization (equal); writing – review and editing (equal). **Ho‐Chang Kuo:** Data curation (equal); funding acquisition (equal); investigation (equal); methodology (equal); project administration (equal); supervision (equal); writing – review and editing (equal).

## CONFLICT OF INTEREST STATEMENT

All authors declare no biomedical financial interests or potential conflicts of interest.

## ETHICS STATEMENT

This study was approved by the local Ethics Committee of the Chang Gung Memorial Hospital (reference number 201900465A3). Written informed consents were obtained from all the enrolled children and their parents or guardians.

## Data Availability

The data are available within the paper from the manuscript's corresponding author on reasonable request.
